# Supplementary figures and images for: Macrophage Inhibitor Clodronate Enhances Liver Transduction of Lentiviral but Not Adeno-Associated Viral Vectors or mRNA Lipid Nanoparticles in Neonatal and Juvenile Mice
Source: Cells. 2024 Nov 29;13(23):1979. doi: 10.3390/cells13231979 (PMC11640373; doi:10.3390/cells13231979)

Supplementary Figure S2. Uncropped blots for LNP-mRNA mediated liver transduction

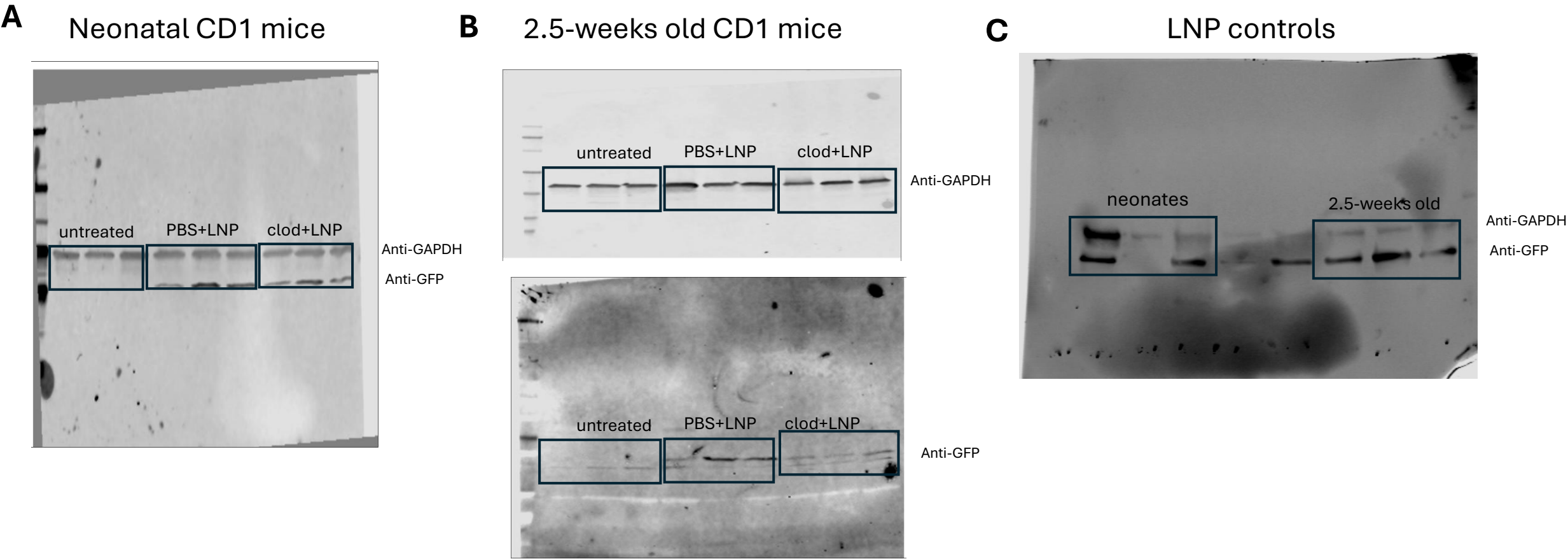

Supplement: Supplementary file 1 [file cells-13-01979-s001.zip › supplementary figure s2.pdf]
